# Interactive effects of body mass index and physical activity on sleep quality in nursing students

**DOI:** 10.3389/fpsyt.2025.1643841

**Published:** 2025-09-03

**Authors:** Guangjian Li, Mengying Wu, Guo Lu, Zijun Yu, Zhigang Bao, Chengying Yu, Jingping Shu, Jinmei Zhu, Xugui Sun

**Affiliations:** ^1^ Graduate School, Wannan Medical College, Wuhu, Anhui, China; ^2^ Psychology Department, Changzhou Dean Hospital, Changzhou, Jiangsu, China; ^3^ Pharmacy Department, The Second People’s Hospital WuHu, Wuhu, Anhui, China; ^4^ School of Basic Medical Sciences, Liaoning University of Traditional Chinese Medicine, Shenyang, Liaoning, China; ^5^ College of Humanities and Management, Wannan Medical College, Wuhu, Anhui, China; ^6^ College of Nursing, Xuancheng Vocational & Technical College, Xuancheng, Anhui, China; ^7^ Rehabilitation Department, Changzhou Dean Hospital, Changzhou, Jiangsu, China; ^8^ Physical Education Teaching and Research Office, Wannan Medical College, Wuhu, Anhui, China

**Keywords:** nursing students, body mass index, physical activity, sleep disorders, interaction

## Abstract

**Background:**

Sleep problems are highly prevalent among nursing students, who often experience academic stress, irregular schedules, and heavy clinical training, which may negatively affect their physical and mental health as well as learning outcomes. Body mass index (BMI) and physical activity are recognized as important factors associated with sleep quality, but their combined effects in nursing students remain insufficiently explored. This study examined the effects of BMI and physical activity on sleep quality in nursing students and explored their interaction, aiming to improve this population’s lifestyle and sleep health.

**Methods:**

A cross-sectional study was conducted on 1,746 nursing students from six universities in Anhui and Jiangsu provinces, China. Data were collected using a general information questionnaire, the Self-Rating Scale of Sleep (SRSS), the Physical Activity Rating Scale (PARS-3), and BMI measurements. Logistic regression models were employed to identify influencing factors and analyze the interaction between BMI and physical activity.

**Results:**

The positive screening rate for sleep disorders in nursing students was 38.1%. Logistic regression analysis showed that internship experience (OR = 1.282, 95% confidential interval, or CI: 1.003–1.639) and low-intensity physical activity (OR = 5.820, 95% CI: 3.408–9.942) were risk factors for sleep disorders. Conversely, being underweight or normal weight (OR = 0.456, 95% CI: 0.303–0.687) and overweight (OR = 0.436, 95% CI: 0.269–0.704) were protective factors. Interaction analysis revealed significant multiplicative interactions between overweight and engagement in moderate-to-high intensity physical activity (OR = 6.582, 95% CI:1.670–25.943) and between obesity and engagement in moderate-to-high intensity physical activity (OR = 6.231, 95% CI:1.518–25.575). While additive interaction indicators did not reach statistical significance, a synergistic trend was observed.

**Conclusion:**

The prevalence of sleep disorders in nursing students is relatively high. BMI and physical activity interact and are both significant influencing factors. Thus, comprehensive interventions targeting BMI and physical activity should be emphasized in nursing students’ health management to improve their sleep quality and overall well-being.

## 1 Introduction

Sleep is a vital physiological process for maintaining physical functioning, cognitive performance, and overall health (1). Sleep problems are common among college students, with reported prevalence rates ranging from 30.4% to 59.1% in different countries (2). In China, recent studies have shown that 35.65–72.3% of nursing students experience poor sleep quality, which is higher than the prevalence reported in the general college student population (3, 4). This high prevalence is associated with negative academic outcomes, impaired clinical performance, and increased psychological distress, and may also lead to long-term fatigue, reduced alertness, memory decline, various physical illnesses, as well as psychological issues such as depression, anxiety, and suicidal ideation (5, 6). Further, sleep quality is a key factor affecting college students’ physical and mental health and academic performance (7). Given its significant impact, sleep quality is particularly important for nursing students, as it not only influences their learning ability and clinical performance but also affects their long-term occupational health. However, due to their intense academic course loads, irregular schedules during clinical internships, and elevated psychological stress, nursing students are at a higher risk of developing sleep disorders (8, 9).

Body mass index (BMI) is a commonly used indicator to assess an individual’s weight status and has been widely applied in studies exploring its impact on health (10). Previous research has revealed the complex relationship between BMI and sleep quality, with both high and low BMI being associated with sleep problems. For example, obesity may increase the risk of obstructive sleep apnea (OSA) (11), and being underweight may contribute to fragmented sleep (12). In addition, physical activity is considered an important behavioral factor in improving sleep quality. Moderate exercise can enhance deep sleep, shorten sleep onset latency, and reduce nighttime awakenings (13). However, it remains unclear whether the effect of physical activity on sleep differs across BMI categories and whether BMI and physical activity interact to influence sleep quality.

Previous studies have primarily examined the independent effects of BMI or physical activity on sleep quality, with limited attention to their potential interaction. Moreover, few studies have specifically targeted nursing students, despite their unique academic demands, irregular schedules during clinical internships, and elevated psychological stress that may collectively increase their vulnerability to sleep disorders. Most existing research has been conducted in single institutions with relatively small sample sizes, which may limit the generalizability of the findings. The present multi-center study addresses these gaps by examining both multiplicative and additive interactions between BMI and physical activity in predicting sleep disorders among nursing students in China, thereby providing more comprehensive evidence for targeted interventions in this high-risk group.

Therefore, this study investigated the effects of BMI and physical activity on sleep quality in nursing students and to analyze their interaction. By conducting a cross-sectional survey, we identified differences in physical activity participation in students with varying BMI levels and explored how these factors jointly influence sleep quality. The results provide scientific evidence for optimizing lifestyle and health management strategies in nursing students, thus improving their overall health and academic performance.

## 2 Methods

### 2.1 Study participants

Nursing students from six universities in Anhui and Jiangsu provinces were recruited as study participants.

Inclusion criteria were as follows: 1) enrolled as nursing students; 2) neurotypical, able to communicate effectively, and no reading difficulties; and 3) willing to participate in the study.

Exclusion criteria included the following: 1) diagnosed with mental illness; 2) an intellectual disability; and 3) a severe physical illness.

The sample size was calculated based on previous literature using the formula for cross-sectional studies: *N=[Z ^2^_α/2_ π (1−π)]/δ ^2^*, an average prevalence of sleep disorders among Chinese nursing students of 35.65% (*π* = 0.3565) was used (4), with a 95% confidence level (*Z* = 1.96) and an absolute precision of 3% (*δ* = 0.03). This yielded a minimum sample size of 979, which increased to 1,088 after allowing for a 10% non-response rate. Considering the multi-center, class-based clustered design, we applied a design effect (DEFF) of 1.45–1.50, resulting in a target sample size of approximately 1,578–1,632. The final valid sample of 1,746 nursing students exceeded this target, ensuring robust statistical power for overall analyses and cross-tabulations.

### 2.2 Survey instruments

#### 2.2.1 General information questionnaire

Demographic data were collected using a researcher-designed questionnaire, which included variables such as gender, age, academic year, place of residence, educational track, leadership role within student organizations, and whether the participant was from the single-child family.

#### 2.2.2 Self-Rating Scale of Sleep

The SRSS, developed by Professor Jianming Li, can be used to screen individuals with sleep problems across various populations and can also be used to evaluate treatment efficacy before and after intervention. The instrument comprises 10 items, with responses rated on a five-point Likert scale ranging from 1 to 5, with higher ratings indicating more severe sleep disturbances. Total scores range from 10 (no sleep problems) to 50 (severe sleep problems). Based on previous research, a total score of ≥23 was defined as “SRSS-positive”, indicating the presence of sleep problems (14, 15).

#### 2.2.3 Physical Activity Rating Scale-3

The PARS-3, originally developed by Japanese scholar Hashimoto and later revised by Chinese scholar Deqing Liang, is used to assess the intensity and frequency of physical activity. The physical activity score was calculated using the formula: intensity × (duration – 1) × frequency. Each of the three dimensions (intensity, duration, and frequency) was rated on a five-point Likert scale ranging from 1 to 5, resulting in a total score ranging from 0 to 100. Scores ≤19 indicate low physical activity; 20–42, moderate activity; and ≥43, high activity. The Cronbach’s α coefficient of the scale among Chinese college students is 0.796 (16, 17).

#### 2.2.4 Body mass index

Body mass index was calculated using participants’ measured height and weight according to the formula: BMI=weight (kg)/height² (m²). Following the Guidelines for the Prevention and Control of Overweight and Obesity in Chinese Adults, BMI values were classified as follows: underweight/normal weight (BMI ≤ 23.9 kg/m²), overweight (BMI 24.0–27.9 kg/m²), and obesity (BMI ≥28.0 kg/m²) (18).

#### 2.2.5 Ethical statements

The study was reviewed and approved by the Ethics Committee of Wannan Medical College, Wuhu, China (Approval No (2023). Ethics Review No. 206). All of the participants provided informed consent prior to participation.

### 2.3 Data collection

Data were collected between January 2024 and December 2024 via an online survey platform. Before data collection, the research team contacted designated faculty members at each of the six participating universities to obtain permission and coordinate the distribution of the questionnaire link. The faculty members then sent the online survey link to eligible nursing students through class communication groups on widely used Chinese messaging applications. On the first page of the online survey, participants were informed of the study purpose, procedures, and confidentiality measures, and electronic informed consent was obtained prior to accessing the questionnaire. Each IP address was restricted to a single submission to prevent duplicate responses. To ensure data quality, questionnaires completed in less than 100 seconds or with evident logical inconsistencies were excluded. In total, 1,800 questionnaires were distributed, and 1,746 valid responses were retained after quality control, yielding a 97.0% valid response rate.

### 2.4 Statistical analysis

Statistical analysis was performed using SPSS version 26.0 and R version 4.2.1. Categorical variables were described using frequencies and percentages, with comparisons performed via the chi-square (χ²) test. For continuous variables, normality was assessed prior to analysis. Normally distributed data were presented as mean ± standard deviation and compared using independent-samples t-tests. Non-normally distributed data were reported as medians with interquartile ranges (IQR) and compared using the Mann–Whitney U test. Logistic regression models were used to identify factors associated with sleep disorder positivity. To assess multiplicative interactions between BMI categories and physical activity levels, the researchers employed logistic regression models. Additive interaction was assessed by calculating the Relative Excess Risk due to Interaction (RERI), Attributable Proportion due to Interaction (API), and Synergy Index (SI). Interaction was considered statistically significant if the 95% confidence interval (CI) for RERI and API did not include 0 and for SI did not include 1.

## 3 Results

### 3.1 General information

A total of 1,746 nursing students were included in this study, of whom 313 (17.9%) were male and 1,433 (82.1%) were female. Among these, 221 (12.7%) were undergraduates, and 1,525 (87.3%) were diploma (associate degree) students. A total of 343 participants (19.6%) had clinical internship experience. The distribution by academic year was as follows: 1,217 (69.7%) freshmen, 266 (15.2%) sophomores, 179 (10.3%) juniors, and 84 (4.8%) seniors. A total of 520 students (29.8%) hailed from urban areas, and 1,226 (70.2%) were from rural areas. There were 478 (27.4%) student leaders and 384 (22.0%) from single-child family. Details are presented in **Table 1**.

**Table 1:** Comparison of sleep disorder positivity rates in nursing students by demographic characteristics.

Variable	Category	Sample Size n (%)	Sleep Disorder Positive (n=665)	*χ^2^/t*	*P*
Sex				3.335	0.068
	Male	313 (17.9)	105		
	Female	1433 (82.1)	560		
Education Level				7.255	0.007
	Undergraduate	221 (12.7)	66		
	Diploma	1525 (87.3)	599		
Academic Year				2.276	0.517
	Freshman	1217 (69.7)	453		
	Sophomore	266 (15.2)	103		
	Junior	179 (10.3)	77		
	Senior	84 (4.8)	32		
Place of Origin				0.108	0.742
	Urban	520 (29.8)	195		
	Rural	1226 (70.2)	470		
Internship Experience				5.768	0.016
	Yes	343 (19.6)	150		
	No	1403 (80.4)	515		
Student Leader				2.082	0.149
	Yes	478 (27.4)	169		
	No	1268 (72.6)	496		
Single-Child Family				0.256	0.613
	Yes	384 (22.0)	142		
	No	1362 (88.0)	523		
BMI				10.964	0.004
	Underweight/Normal	1405 (80.5)	525		
	Overweight	231 (13.2)	82		
	Obese	110 (6.3)	58		
Physical Activity Level				52.323	0.000
	Low	1596 (91.4)	649		
	Moderate to High	150 (8.6)	16		
Age (years)		18.84 ± 0.96	18.88 ± 1.06	−0.768	0.443

### 3.2 Comparison of positive rates for sleep disorders in nursing students

In this study, a total of 665 nursing students were screened positive for sleep disorders, yielding a positive screening rate of 38.1%. The difference in the positive rates of sleep disorders between undergraduate and vocational college students was statistically significant (*P*<0.05). Students with internship experience had a higher positive rate of sleep disorders (*P*<0.05). Differences existed in the positive rates of sleep disorders among different BMI categories (*P*<0.05), as well as among different levels of physical activity intensity (*P*<0.05). See **Table 1** for details.

### 3.3 Logistic regression analysis of risk factors for sleep disorders in nursing students

Variables that showed significant differences in univariate analysis were included as independent variables, while the sleep disorder status (positive =1, negative =0) was used as the dependent variable in a multivariate logistic regression analysis. Internship experience (OR = 1.282, 95% CI: 1.003–1.639, *P* = 0.047) and low-intensity physical activity (OR = 5.820, 95% CI: 3.408–9.942, *P* < 0.001) were independent risk factors for sleep disorders. In contrast, underweight or normal weight (OR = 0.456, 95% CI: 0.303–0.687, *P* < 0.001) and overweight (OR = 0.436, 95% CI: 0.269–0.704, *P* < 0.001) were protective factors. See **Table 2** for details.

**Table 2:** Multivariate logistic regression analysis of risk factors for sleep disorders.

Factor	*B*	*SE*	*Wald χ^2^*	*P*	*OR*	*95% OR*
Internship (Ref = No)	0.249	0.125	3.956	0.047	1.282	1.003–71.639
BMI (Ref = Obese)						
Underweight/Normal	−0.785	0.209	14.137	0.000	0.456	0.303–0.687
Overweight	−0.831	0.245	11.494	0.000	0.436	0.269–0.704
Physical Activity (Ref = Moderate-to-High)						
Low intensity	1.761	0.273	41.577	0.000	5.820	3.408–9.942
Education Level (Ref = Diploma)	−0.230	0.163	2.006	0.157	0.794	0.578–1.092
Constant	−1.421	0.320	19.718	0.000	0.241	

### 3.4 Multiplicative interaction between BMI and physical activity in predicting in nursing students

To explore the combined effect of BMI and physical activity on sleep disorders, we constructed a multivariate logistic regression model including BMI categories (overweight, obese), physical activity level (moderate-to-high), and their interaction terms. After adjusting for confounders, we observed significant multiplicative interactions: overweight × moderate-to-high physical activity: OR = 6.582 (95% CI: 1.670–25.943, *P* = 0.007); obese × moderate-to-high physical activity: OR = 6.231 (95% CI: 1.518–25.575, *P* = 0.011). These results indicate the synergistic effect of BMI and physical activity on sleep disorders. See **Table 3** for details.

**Table 3:** Multiplicative interaction between BMI and physical activity in predicting sleep disorders.

Variable	*Unadjusted OR*	95% *CI*	*P*	*Adjusted OR*	95% *CI*	*P*
Overweight	0.884	0.653–0.749	0.425	0.892	0.658–1.210	0.463
Obese	1.893	1.238–2.893	0.003	1.959	1.274–3.012	0.002
Moderate-to-High PA	0.072	0.029–0.177	<0.001	0.077	0.031–0.190	<0.001
Overweight × Moderate-to-High PA	6.193	1.579–24.286	0.009	6.582	1.670–25.943	0.007
Obese × Moderate-to-High PA	5.994	1.471–24.434	0.012	6.231	1.518–25.575	0.011

### 3.5 Crossover analysis of BMI and physical activity on sleep disorder risk in nursing students

Using lean and normal-weight nursing students who engaged in low-intensity physical activity as the reference group, the results of the univariate logistic regression analysis showed that obese nursing students with low-intensity activity had a 1.893-fold higher risk of sleep disorders (OR = 1.893, 95% CI: 1.238–2.893). In contrast, lean and normal-weight nursing students who engaged in moderate- to high-intensity activity had markedly lower odds, reduced to 7.2% of that in the reference group (OR = 0.072, 95% CI: 0.029–0.177). After adjusting for potential confounders in the multivariate logistic regression analysis, the risk for obese students with low-intensity activity increased to 1.959-fold that of the reference group (OR = 1.959, 95% CI: 1.277–3.012). Similarly, lean and normal-weight students with moderate- to high-intensity activity maintained a significant protective effect, with odds reduced to 7.7% of that in the reference group (OR = 0.077, 95% CI: 0.031–0.190). See **Table 4** for details.

**Table 4:** Crossover analysis of BMI and physical activity on sleep disorder risk.

BMI	PA Level	n	Sleep Disorders n (%)	P	Crude *OR* (95% CI)	P	Adjusted *OR* (95% CI)
Underweight/Normal	Low	1296	520 (40.1%)	–	1	–	1
Underweight/Normal	Moderate-to-High	109	5 (4.6%)	<0.001	0.072 (0.029–0.177)	<0.001	0.077 (0.031–0.190)
Overweight	Low	207	77 (37.2%)	0.425	0.884 (0.653–1.197)	0.463	0.892 (0.658–1.210)
Overweight	Moderate-to-High	24	5 (20.8%)	0.065	0.393 (0.146–1.058)	0.117	0.449 (0.165–1.223)
Obese	Low	93	52 (55.9%)	0.003	1.893 (1.238–2.893)	0.002	1.959 (1.277–3.012)
Obese	Moderate-to-High	17	6 (35.3%)	0.687	0.814 (0.299–2.215)	0.895	0.934 (0.339–2.576)

### 3.6 Additive interaction analysis between BMI and physical activity in nursing students

We calculated additive interaction indices to further assess the joint effects of BMI and physical activity on sleep disorder risk. The results showed a potential positive additive interaction between obesity and moderate-to-high physical activity, albeit not a statistically significant one: RERI = 0.361 (95% CI: −0.144 to 0.866); AP = 0.248 (95% CI: −0.002 to 0.498); Synergy Index (S) =4.845 (95% CI: 0.353–66.457). These results indicate a possible synergistic effect between obesity and moderate-to-high physical activity levels on sleep disorder outcomes in nursing students.

## 4 Discussion

This study found that the positive screening rate for sleep disorders in nursing students rose as high as 38.1%, which is notably higher than the rates reported by Jin Yaping et al. (28.5%) and Li Yusi et al. concerning nursing interns (31.65%) (19, 20). It is also higher than the 22.7% reported by Wang Xiaokang et al. concerning medical students in Tianjin (21). These results indicate that nursing students are a high-risk group for sleep disorders. This result may be due to the unique academic and lifestyle challenges they face, such as their heavy academic workload, emotional stress, and irregular schedules (22, 23).

Logistic regression analysis showed that internship experience was a significant risk factor for sleep disorders. Specifically, nursing students with internship experience had a 1.282-fold higher risk of developing sleep disorders compared with those without such experience. This result may be due to the demands of clinical internships, which often involve shift work, stressful interpersonal communication, emotional strain, and an insufficient mastery of disease-related knowledge—factors that can induce anxiety and disrupt circadian rhythms, ultimately impairing sleep quality (24). The results ultimately highlight the need for universities and internship institutions to monitor the physical and mental health of nursing students during internships, especially by providing more empathetic scheduling and psychological support systems.

This study also found that lower levels of physical activity were significantly associated with a higher risk of sleep disorders, which is consistent with the results of Fei et al. (25) Compared with students engaging in moderate-to-high-intensity physical activity, those with low physical activity levels had a 5.82-fold increased risk of sleep disorders. This result aligns with previous research demonstrating that regular physical activity can improve sleep by regulating melatonin secretion, enhancing sleep architecture, and alleviating anxiety symptoms (26–28). Although students with moderate-to-high physical activity made up only 8.6% of the sample, their rate of positive screening for sleep disorders was considerably lower, underscoring the practical value of promoting physical exercise in nursing students.

BMI was also identified as an important factor influencing sleep disorders. Compared with the obese group, students with underweight or normal BMI (OR = 0.456) and those who were overweight (OR = 0.436) had a significantly lower risk of sleep disorders. The link between obesity and sleep disorders can be explained by multiple physiological mechanisms. First, fat accumulation in obese individuals may narrow the upper airway, leading to conditions such as obstructive sleep apnea, which can disrupt sleep continuity (29). Second, obesity is often accompanied by chronic inflammation and insulin resistance, which may both affect sleep regulation through the hypothalamic–pituitary–adrenal axis (30, 31).

This study identified a significant multiplicative interaction between BMI and physical activity levels in relation to sleep quality, indicating that these factors do not act independently but rather interact synergistically. Specifically, moderate-to-high physical activity significantly attenuated the risk of sleep disorders among overweight or obese individuals. The interaction terms for overweight × moderate-to-high-intensity activity (OR = 6.582) and obesity × moderate-to-high-intensity activity (OR = 6.231) reflected a strong protective effect. Mechanistically, regular moderate- to high-intensity exercise can reduce visceral fat and help control body weight, thereby alleviating obesity-related sleep disturbances such as obstructive sleep apnea and snoring (32). It can also enhance insulin sensitivity, stabilize blood glucose levels (33), reduce chronic low-grade inflammation (34), and relieve anxiety and depression (35), all of which contribute to better sleep quality. These findings suggest that increasing physical activity could buffer the adverse impact of high BMI on sleep quality, providing theoretical support for targeted exercise interventions in this population. In our additive interaction analysis, although the relative excess risk due to interaction (RERI) and attributable proportion (AP) were not statistically significant, the synergy index (S = 4.845) indicated a potential synergistic effect, implying that combined weight management and physical activity interventions may yield greater benefits, particularly for obese nursing students.

Our results also showed that internship experience and low physical activity levels are significant risk factors for sleep disorders in nursing students. Reduced volume and intensity of physical activity were associated with higher risks, suggesting that moderate-to-high-intensity exercise plays a crucial role in maintaining sleep quality. However, nursing students—especially those in clinical internships—may experience decreased activity due to time constraints. According to the American College of Sports Medicine, adolescents should engage in at least 60 minutes of combined moderate- and high-intensity activity daily (36). For overweight individuals, gradual increases in duration and intensity are recommended to enhance adherence and safety (37). Evidence shows that two or three supervised sessions plus one or two unsupervised sessions per week can yield substantial health benefits over six months or longer (38–40). To ensure safety, adequate warm-up and cool-down routines are essential, and obese students should undergo regular health checks and receive exercise-related education. For this group, 60–90 minutes of exercise per session, including approximately 10 minutes of warm-up and cool-down, at a frequency of three to five sessions per week, is recommended, tailored to individual physical conditions (41).

The study results also highlight the protective effects of underweight or normal BMI and overweight status—compared with obesity—on sleep quality in nursing students, indicating that higher BMI may adversely affect sleep. These results emphasize the importance of weight management in maintaining the health of nursing students and suggest that both individuals and institutions should be aware of the implications of BMI. Adopting healthy dietary habits and engaging in regular exercise to keep BMI within an optimal range may help prevent sleep problems. For those who have sleep disorders, interventions should consider BMI as part of a comprehensive health management plan aimed at improving both sleep quality and overall well-being.

Moreover, the identified interaction between BMI and physical activity highlights that student with higher BMI may be more vulnerable to the adverse effects of insufficient physical activity, which in turn, increases their risk of developing sleep disorders. This result underscores the necessity of integrating weight management with physical activity promotion as a health strategy for nursing students. Moderate to high-intensity physical activity not only improves sleep but may also assist in weight control, thereby reducing obesity-related health risks (38). Educational institutions should thus strengthen health education programs, especially those focused on the importance of physical exercise and BMI management, to enhance students’ awareness and engagement in healthy lifestyle behaviors. Future studies should explore the optimal types and intensities of physical activity for nursing students in different BMI categories and develop personalized exercise programs to effectively improve sleep outcomes.

The strengths of this study include its focus on a special population of nursing students, the use of a multicenter design involving multiple universities across two provinces, a relatively large sample size, and a clearly defined, replicable methodology—with standardized inclusion and exclusion criteria, validated measurement tools, and a uniform online data collection process administered via the Questionnaire Star platform. However, several limitations should also be noted. Firstly, the cross-sectional design restricts the capacity to deduce causal relationships between body mass index, physical activity, and sleep quality. It is recommended that longitudinal or experimental studies be conducted to more firmly establish causal connections. Secondly, although previous studies have suggested that underweight status may be associated with fragmented sleep, we combined underweight and normal weight into a single category because the proportion of underweight participants was small. This combination was necessary to maintain statistical stability but prevented us from examining potential differences between these groups. Thirdly, moderate- and high-intensity physical activity were combined into one category in our analysis due to the small number of students engaging in high-intensity activity. While this approach ensured sufficient statistical power, it also limited our ability to assess potential differences between moderate and high intensity levels. Future research with larger and more diverse samples is warranted to clarify these distinctions. Fourthly, sleep quality and physical activity were evaluated using self-reported questionnaires, which are prone to recall and social desirability biases. These subjective methods may not precisely reflect actual behaviors. Future research should consider incorporating objective assessment tools, such as actigraphy, wearable fitness trackers, or sleep monitoring devices, to improve data reliability. Finally, while participants were recruited from various universities across two provinces, the results may not be applicable to all student populations, particularly those in different regions or educational systems. Moreover, several potential confounding variables—including psychological stress levels, dietary habits, caffeine and alcohol consumption, and screen time—were not measured or controlled for, which could have affected the observed associations. To overcome these limitations, future studies should employ multi-regional or nationwide sampling strategies, control for a wider range of confounders, and utilize longitudinal or interventional designs in conjunction with objective measurement tools to validate and extend these findings.

## Data Availability

The raw data supporting the conclusions of this article will be made available by the authors, without undue reservation.
